# A Two-Phage Cocktail Modulates Gut Microbiota Composition and Metabolic Profiles in an Ex Vivo Colon Model

**DOI:** 10.3390/ijms26062805

**Published:** 2025-03-20

**Authors:** Sthefhany Nohemí Rodríguez-Arellano, Jean Pierre González-Gómez, Bruno Gomez-Gil, Marisela González-Ávila, Juan Ramón Palomera-Hernández, Elisa Barrón-Cabrera, Marcela de Jesús Vergara-Jiménez, Cristobal Chaidez

**Affiliations:** 1 Facultad de Ciencias de la Nutrición y Gastronomía, Universidad Autónoma de Sinaloa, Culiacan 80019, Sinaloa, Mexico; 2 Laboratorio Nacional para la Investigación en Inocuidad Alimentaria (LANIIA), Centro de Investigación en Alimentación y Desarrollo, A.C. (CIAD), Culiacan 80110, Sinaloa, Mexico; 3 Centro de Investigación en Alimentación y Desarrollo, A.C. (CIAD), Unidad Mazatlán en Acuicultura y Manejo Ambiental, Mazatlan 82112, Sinaloa, Mexico; 4 Medical and Pharmaceutical Biotechnology, Centro de Investigación y Asistencia en Tecnología y Diseño del Estado de Jalisco, A.C. (CIATEJ), Guadalajara 44270, Jalisco, Mexico

**Keywords:** microbiota modulation, phage therapy, metabarcoding 16S rRNA, short-chain fatty acid, bacteriophage

## Abstract

Bacteriophage therapy is a promising approach for targeting antibiotic-resistant bacteria and modulating gut microbiota in metabolic diseases such as obesity. This study evaluated the impact of a two-phage cocktail on an ex vivo colonic simulation model of gut microbiota derived from obese individuals, both in its normalized state and after enrichment with *Enterobacter cloacae*, an obesity-related bacteria. Microbiological analyses confirmed that the phage cocktail remained active throughout the colonic regions over three digestion cycles and effectively reduced enterobacterial populations in the enriched microbiota. Metabarcoding of the 16S rRNA gene revealed that phage therapy did not significantly alter the abundance of dominant genera, but selectively reduced *E. cloacae* across all colonic regions. Alpha diversity was significantly affected only in the enriched microbiota, while beta diversity analysis indicated significant compositional shifts during therapy, with reduced dispersion in the final treatment stage. Short-chain fatty acid profiling demonstrated region- and group-specific metabolic responses, with increased lactic and butyric acid concentrations in the ascending colon of the enriched microbiota following phage treatment. This study provides the first ex vivo evidence that a two-phage cocktail can selectively eliminate *E. cloacae* while preserving overall microbiota structure and functionality. These findings establish a foundation for future in vivo studies exploring the role of phage therapy in reshaping gut microbial communities and metabolic profiles, highlighting its potential as a precision tool for managing gut dysbiosis in metabolic disorders.

## 1. Introduction

The human gut harbors an immense microbial population—over 100 trillion microorganisms—including viruses, yeasts, and predominantly bacteria [1,2]. This complex community, known collectively as the gut microbiome, is essential for various physiological functions, from metabolic cross-feeding to immune system modulation, underscoring its vital role in maintaining overall health [3,4]. Disruptions in this microbial balance, known as intestinal dysbiosis [5], have been increasingly associated with the onset of metabolic diseases such as inflammatory bowel disease [6], diabetes [7], and obesity [8], the latter representing a significant global health challenge. Emerging evidence points to endotoxins, particularly lipopolysaccharides (LPS) produced by gut bacteria, as key contributors to obesity through the promotion of inflammation [9]. Notably, LPS from enterobacteria exhibits significantly higher endotoxin activity than those from more abundant gut bacteria like those in the phylum Bacteroidetes [10], highlighting their potential role in obesity and related metabolic disorders. Given these findings, there is a pressing need to develop strategies for modulating the gut microbiota and further clarifying its role in these diseases.

Various strategies have been explored to shape the gut microbiota, including fecal transplantation therapy [11], prebiotics, probiotics, synbiotics, and postbiotics [12], as well as dietary interventions [13]. These approaches primarily aim to enrich beneficial bacteria and restore microbial balance. However, phage therapy offers a distinct mechanism of action, leveraging its high specificity to selectively target and eliminate particular bacterial species or even strains while preserving the overall microbiota composition [14,15]. This specificity allows phage therapies to focus on a narrow group of target microorganisms while maintaining long-term efficacy, as bacteriophages replicate within their bacterial hosts [15]. Additionally, bacteriophages can selectively inhibit pathogenic bacteria without affecting non-target cells, and can even be internalized by mammalian cells for potential metabolic utilization [16]. Recent studies using murine models have shown that phage administration can effectively modulate the gut microbiota and induce metabolic changes [2,4]. Although clinical evidence is still emerging, one study reported that a bacteriophage cocktail targeting *Escherichia coli* in adults increased the commensal bacterium *Bifidobacterium bifidum* [17]. However, both murine and clinical studies require approval from ethics committees, which can prolong timelines and impose methodological limitations based on their recommendations [18].

To overcome the limitations associated with in vivo gut microbiota evaluation, ex vivo colonic fermentation models offer valuable alternatives [19]. Complex dynamic models that simulate the entire digestive system, incorporating both digestion and fermentation processes with continuous nutrient input, have been developed. Notable examples include the SIMulator of the Gastro-Intestinal tract (SIMGI) [19], the Simulator of the Human Intestinal Microbial Ecosystem (SHIME^®^) [20], and the Automatic and Robotic Intestinal System (ARIS) [21]. ARIS is designed to replicate the human gastrointestinal tract across five sections: the stomach, the small intestine, and the ascending, transverse, and descending regions of the colon, where gut microbiota can be cultivated. The system can be customized to study parameters influenced by physiological and dietary factors [21]. Consequently, ARIS serves as an excellent model for evaluating the impact of a bacteriophage cocktail on the composition and diversity of the microbiota in obese patients, analyzing key inflammation-related compounds like short-chain fatty acids (SCFAs), and tracking phage and their host bacteria persistence across different colonic sections, as demonstrated in previous studies [22,23,24]. In this study, we utilized the ARIS model to evaluate the impact of a bacteriophage cocktail on the metabolite profile and gut microbiota composition of obese patients, aiming to explore the potential of phage therapy as an adjunctive strategy for managing metabolic diseases.

## 2. Results

### 2.1. Microbiological Analysis

**Figure 1:**
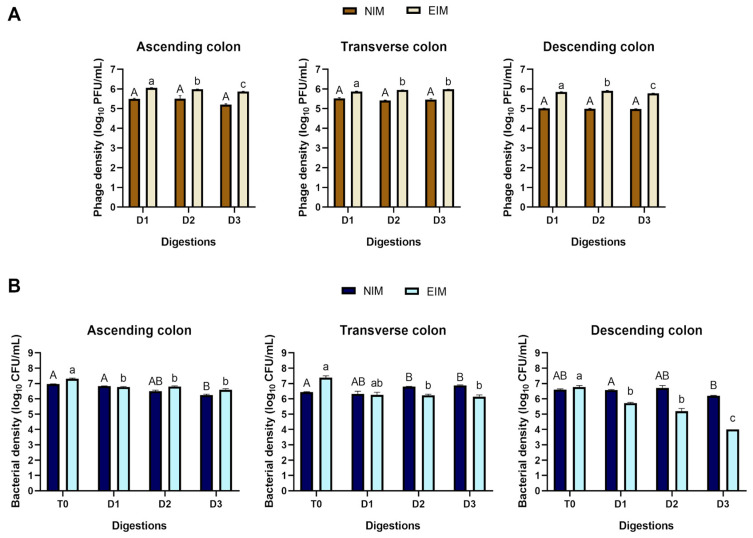
Microbiological analysis of bioreactors simulating colonic regions. (**A**) Bacteriophage titer and (**B**) *Enterobacteriaceae* concentration during the intervention. Different capital letters indicate significant differences (*p* < 0.05) between intervention times in the normalized intestinal microbiota (NIM) of individuals with obesity, while different lowercase letters indicate significant differences between intervention times in the *Enterobacter*-enriched intestinal microbiota (EIM) of individuals with obesity. T0: time 0; D1: digestion 1; D2: digestion 2; D3: digestion 3. Values are the mean of three tests ± standard deviation.

This study was designed to evaluate two distinct groups: the first comprised the normalized intestinal microbiota of individuals with obesity treated with a bacteriophage cocktail. In contrast, the second included the intestinal microbiota of individuals with obesity that had been enriched with *Enterobacter cloacae* ATCC 13047 treated with a bacteriophage cocktail. At the start of the experiment, no bacteriophages targeting *E. cloacae* were detected in the bioreactors simulating the three regions of the colon. Following inoculation with the bacteriophage cocktail (comprising bacteriophages AS5 and AS6 [25]), their persistence was observed throughout the experiment. The bacteriophages maintained consistent activity across the different stages of digestion, remaining unaffected by the simulated digestive conditions of the small intestine (Figure 1).

In the normalized intestinal microbiota (NIM) group, no significant changes in bacteriophage density were observed during digestion in the various regions of the large intestine. Notably, the NIM group exhibited a lower phage density than the *Enterobacter*-enriched intestinal microbiota (EIM) group. Within the EIM group, the highest viral titer was recorded in the ascending colon (AC), reaching 6.11 Log_10_ PFU/mL. However, during digestion stage 3, a significant decline in viral density was observed, decreasing to 5.86 Log_10_ PFU/mL. Conversely, in the transverse colon (TC), a significant increase in viral density was detected, reaching a maximum titer of 5.98 Log_10_ PFU/mL, exceeding the levels recorded in both the ascending (AC) and descending (DC) colonic regions.

Distinct patterns in enterobacterial counts were observed between the two study groups. In the NIM group, enterobacterial abundance remained relatively stable throughout the digestion stages, with significant differences noted in specific regions. The most pronounced change was a reduction of 0.51 ± 0.09 Log_10_ CFU/mL in the descending colon during digestion 3. Conversely, the EIM group displayed a higher initial abundance of enterobacteria at time 0, particularly in the TC. As digestion progressed, a substantial and progressive reduction in enterobacterial counts was observed across all regions of the large intestine. The most notable decrease occurred in the DC, where enterobacterial counts dropped up to 2.76 ± 0.05 Log_10_ CFU/mL, compared to control (time 0), by the end of the digestion stages.

### 2.2. Production of Short-Chain Fatty Acids

SCFAs are essential mediators of beneficial cross-feeding among commensal gut bacteria, promoting colonization resistance and competitive exclusion of pathogens [26]. To assess the impact of phage therapy on the production of these vital metabolites, we quantified the concentrations of acetic, propionic, and butyric acids in the different colonic regions at baseline and after the completion of the phage therapy.

**Figure 2:**
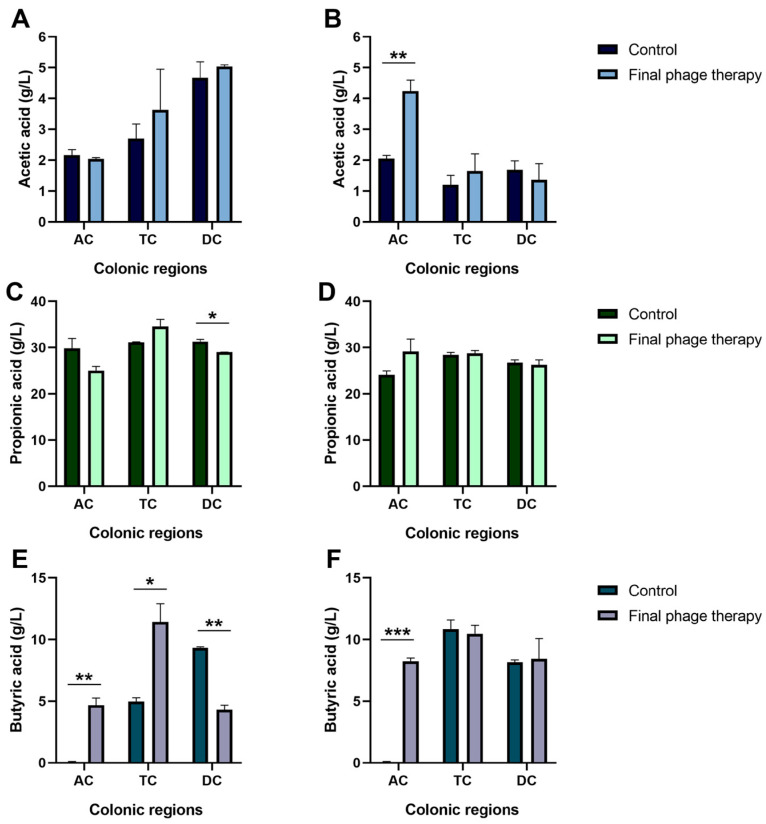
Short-chain fatty acid concentrations in colonic regions of normalized intestinal microbiota of individuals with obesity (**A**,**C**,**E**) and *Enterobacter*-enriched intestinal microbiota of individuals with obesity (**B**,**D**,**F**). AC: ascending colon; TC: transverse colon; DC: descending colon. Values are the mean of three tests ± standard deviation. Significant differences between the control and final phage therapy treatments are indicated as follows: *: *p* < 0.05, **: *p* < 0.01, and ***: *p* < 0.001.

The results revealed significantly higher lactic acid in the AC following phage therapy in the *Enterobacter*-enriched intestinal microbiota. Additionally, butyric acid was detectable exclusively after treatment in this region. In the TC, significant differences were observed only in the normalized intestinal microbiota, with butyric acid concentrations being higher after phage therapy. Lastly, in the descending colon, a decrease in the concentrations of propionic and butyric acids was noted in the NIM group after treatment (Figure 2).

### 2.3. Comparative Analysis of Alpha Diversity

Alpha diversity analysis was performed on the classified sequences to evaluate microbial richness and evenness changes following phage therapy intervention across the different colonic regions. In the NIM group, no significant differences were observed in either the Chao1 or Shannon indexes among the colonic regions, indicating that phage therapy did not affect the richness or evenness of the microbiota analyzed (Table 1).

In contrast, the EIM group exhibited notable differences in the Chao1 index, which primarily reflects microbial richness, between the control, mid-treatment, and post-treatment phases (Table 2). These differences are likely attributable to the initial enrichment with *E. cloacae* at time 0 (control), particularly in the ascending and transverse colons, where values were higher than those observed in the NIM group. This enrichment may have influenced the baseline microbial diversity, resulting in the observed changes during the phage therapy intervention.

### 2.4. Comparative Analysis of Beta Diversity

**Figure 3:**
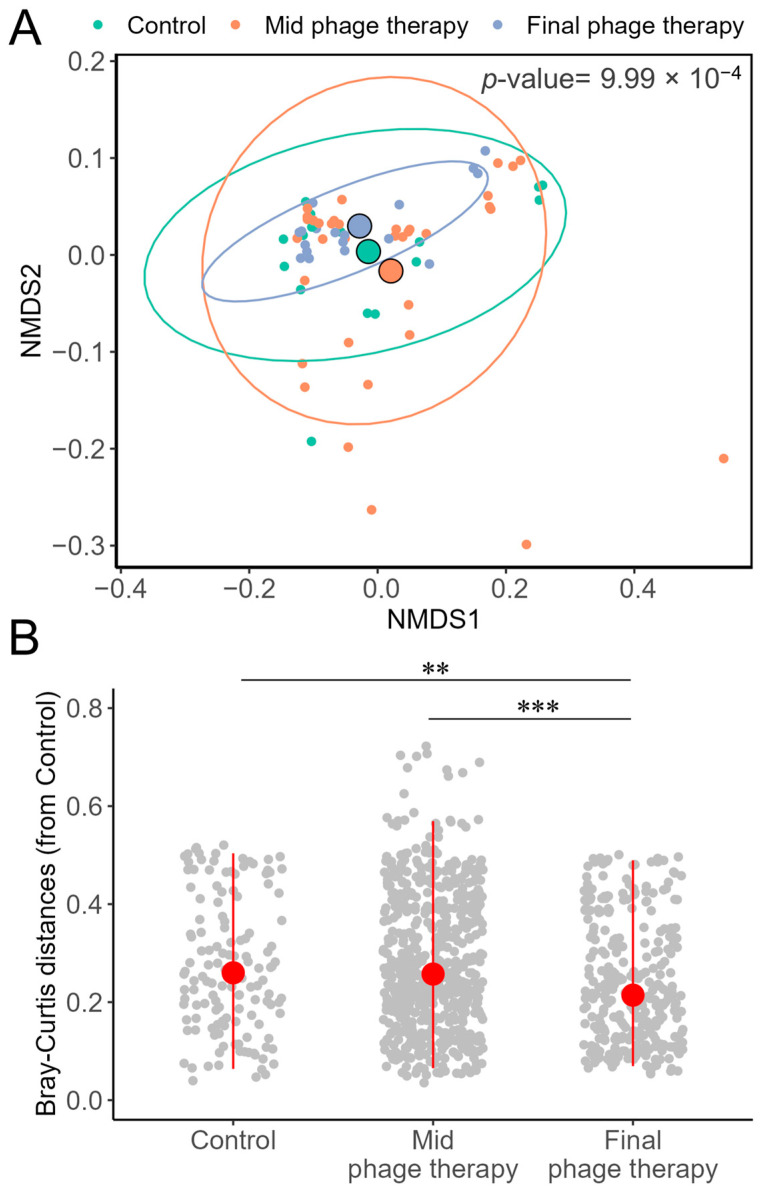
Bray–Curtis dissimilarities of colon microbiota. (**A**) NMDS ordination plot. Delineated circles represent the centroid of each treatment and the *p*-value corresponds to the PERMANOVA calculation. (**B**) Distances from each sample against the control. Significative differences represent: **: *p* < 0.01, and ***: *p* < 0.001; by Wilcoxon pairwise test.

The beta diversity analysis, based on Bray–Curtis distances and visualized through non-metric multidimensional scaling (NMDS), revealed significant differences across the control and phage therapy stages, as determined by PERMANOVA. Notably, microbial communities at the final stage of phage therapy exhibited lower dispersion than those in the control and mid-phage therapy stages, despite the centroids of all groups being closely positioned (Figure 3). Additionally, the dissimilarities of microbial communities at each treatment stage were evaluated relative to the control. This analysis confirmed that the communities at the final phage therapy stage displayed significantly lower dispersion than both the mid-phage therapy and control groups.

### 2.5. Taxonomic Diversity

**Figure 4:**
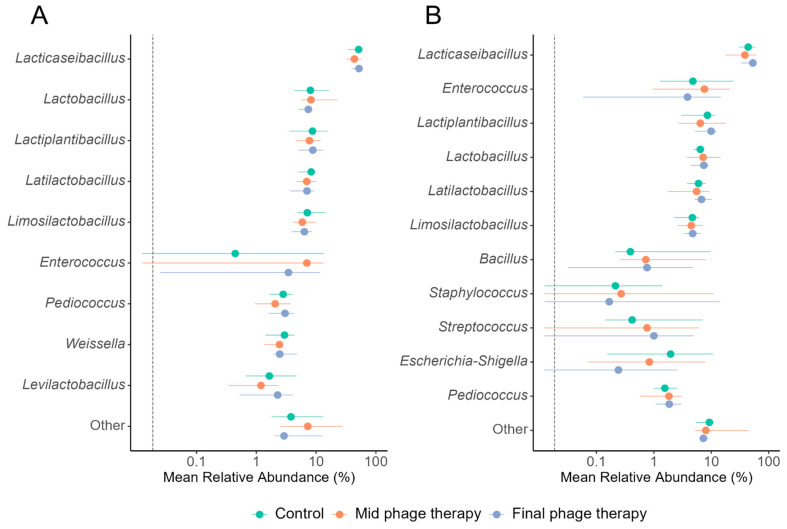
Relative abundance of the major genera detected in the colon. (**A**) Phage therapy in normalized intestinal microbiota of individuals with obesity. (**B**) Phage therapy in *Enterobacter*-enriched intestinal microbiota of individuals with obesity. Colored dots represent the median for each treatment, with lines indicating the treatment range within a 0.95 confidence interval. The vertical dashed line denotes the detection limit. No significant differences were found between genera mean relative abundance by the Kruskal–Wallis test.

Taxonomic assignment of the 16S rRNA sequences enabled an evaluation of the relative abundance of bacterial genera to determine the impact of phage therapy on specific groups. In both study groups, the dominant genera were primarily lactic acid bacteria, particularly *Lacticaseibacillus*. Enterobacteria were exclusively detected in the EIM group, represented by the *Escherichia-Shigella* cluster with a relative abundance of approximately 1% (Figure 4). This grouping could not be resolved further due to their close evolutionary relationship. A Kruskal–Wallis non-parametric test revealed no significant differences in the relative abundance of the main detected genera between treatments in either the NIM or EIM samples. These findings indicate that phage therapy did not alter the composition of the predominant bacterial genera in the colon microbiota.

### 2.6. Enterobacter cloacae 16S rRNA Gene Sequences

Since *Enterobacter* was not identified as one of the most abundant genera in the general taxonomic diversity analysis, we precisely mapped the sequences of each sample against the 16S rRNA gene of *E. cloacae* ATCC 13047 to evaluate changes in its abundance. In the normalized microbiota of individuals with obesity treated with phage therapy, fewer than two sequences were mapped across the three colonic regions at any time. Conversely, a significantly higher abundance of these sequences was observed in the *Enterobacter*-enriched microbiota, particularly in the control samples immediately after enrichment. Among the colonic regions, the transverse colon exhibited the highest concentration of these sequences (Table 3). As digestion progressed, a marked reduction in the abundance of *E. cloacae* sequences was recorded across all colonic regions. These findings suggest that phage therapy effectively reduced the density of this bacterial genus throughout the colon in this experimental model.

## 3. Discussion

The gut microbiota plays a crucial role in maintaining homeostasis within the organism. Its interactions with the enteric nervous system and the brain influence appetite, digestion, metabolism, and immune function, all of which are key factors in developing metabolic disorders such as obesity [8]. Individuals with obesity, often associated with high-fat diets, exhibit increased intestinal permeability, allowing LPS to translocate from the intestinal lumen into the bloodstream. This process compromises tight junction proteins in the epithelium, further weakening the intestinal barrier and triggering low-grade chronic inflammation [27,28]. Addressing overweight and obesity requires a multifaceted approach, ranging from lifestyle modifications to advanced interventions. Given the gut microbiota’s essential role in this process, innovative biotherapies targeting dysbiosis have emerged as promising strategies [29]. Fecal microbiota transplantation, probiotics, prebiotics, and phage therapy have all shown potential in modulating the microbiome [30,31]. Among these, phage therapy stands out because it selectively eliminates pathogenic bacteria while preserving beneficial microbial communities. It is a novel tool for restoring gut health and promoting metabolic regulation [32].

Our findings underscore the potential of phage therapy to selectively reduce specific microorganisms in the gut microbiota without disrupting overall diversity while enhancing the uniformity of the native microbiota. Although obesity is a multifactorial condition influenced by both environmental and genetic factors [33], it is well-established that LPS produced by gut bacteria contribute to inflammation and may increase obesity risk [9]. In our study, microbiological analysis revealed a significant reduction in enterobacterial counts in the descending colon when *E. cloacae* was present in the intestinal microbiota. This is particularly relevant as Gram-negative bacteria, including enterobacteria, are major LPS producers, and their reduction may lower susceptibility to colitis and attenuate inflammatory responses mediated by proinflammatory cytokines such as TNF-α and IL-1β [34,35]. Notably, the potent reduction in enterobacteria was only observed in the *Enterobacter*-enriched microbiota (EIM) group, highlighting the importance of direct phage-host interactions in the colon, where replication amplifies the phage’s host-reducing effects [24]. This conclusion is further supported by the observed increase in phage titers throughout all digestion stages in the EIM group, which were approximately tenfold higher than in the normalized microbiota (NIM) group. These results align with findings from previous in vivo [36] and ex vivo [23] studies, reinforcing the efficacy of phage therapy in selectively reducing bacterial populations in the gut microbiota.

One of the most significant findings of our study is the marked reduction in *E. cloacae* sequences following the administration of the phage cocktail. At the same time, the predominant bacterial genera remained statistically unaffected. Similar effects have been documented in clinical trials targeting other pathogens. For instance, Febvre et al. [17] reported that a commercial phage cocktail against *Escherichia coli*, administered for 28 days, led to a significant reduction (*p* = 0.03) in *E. coli* sequences in stool samples compared to a placebo, without significant alterations in alpha or beta diversity, confirming that the intervention did not disrupt the commensal microbiota.

Additionally, several studies have explored the impact of phage therapy on non-communicable metabolic diseases, including sclerosing cholangitis [37], non-alcoholic fatty liver disease [38,39], and inflammatory bowel diseases [24] associated with *Klebsiella pneumoniae* or hepatitis linked to *Enterococcus faecalis* [40]. These studies have demonstrated promising outcomes, with effective suppression of disease-associated bacteria and subsequent improvements in clinical symptoms. However, in other cases, such as *Escherichia coli*-associated gastrointestinal distress, clinical trials have confirmed the safety of phage therapy but found no evidence of therapeutic efficacy [41,42]. Conversely, a preclinical trial investigating phage therapy for adherent invasive *Escherichia coli*-associated Crohn’s disease demonstrated that the treatment was safe, did not disrupt gut microbiota composition, and provided protection against both clinical symptoms and histological signs of inflammation in mice [43].

These findings highlight that the success of phage therapy depends on multiple factors, including the specificity and activity of each phage, as well as the role of the target bacteria in disease progression [44]. Therefore, while phage therapy holds significant potential as a precision tool for microbiota modulation, its efficacy must be assessed on a case-by-case basis, considering microbial dynamics and host-pathogen interactions [45].

SCFAs, including acetic, propionic, and butyric acids, are key metabolites produced through the fermentation of dietary polysaccharides by gut microbiota. SCFAs play critical roles in inducing the expression of intestinal hormones that contribute to appetite suppression, improved glucose tolerance, and enhanced insulin sensitivity. In individuals with obesity, reduced gut microbiota diversity is often associated with diminished SCFAs production, negatively impacting metabolic processes [46,47]. In our study, we observed a trend toward increased SCFAs production following phage cocktail inoculation in both groups. However, this effect varied by colonic region, with a significant increase in butyric acid production observed in the ascending colon, likely influenced by local pH conditions. Butyric acid serves as the primary energy source for colonocytes and helps suppress the migration of pro-inflammatory mediators from resident immune cells [48]. In a study by Laforêt et al. [49] the phage vB_KpnP_K1-ULIP33 targeting *Klebsiella pneumoniae* was evaluated in an ex vivo model of healthy microbiota. Their findings demonstrated microbiota modulation without significant changes in SCFAs production following phage addition, although total SCFAs levels were higher in the distal colon. This suggests that while phages can induce microbiota shifts, they do not necessarily alter SCFAs production, which is crucial for maintaining metabolic and immune functions. Our results emphasize the importance of ensuring that phage therapy does not compromise SCFAs production, as these metabolites are essential for intestinal and systemic health. However, prolonged evaluation is necessary to determine whether these effects persist over time.

Our study highlights the potential of phage therapy as a targeted approach to modulating the gut microbiota without disrupting its overall diversity. Specifically, our results demonstrate that bacteriophage treatment effectively reduces *E. cloacae* in an enriched microbiota model, particularly in the descending colon, while preserving the balance of commensal bacteria. Our findings also emphasize the importance of phage-host interactions in the colon, as the therapeutic effect was most pronounced when the target bacteria were abundant. Furthermore, the observed increase in phage titers suggests successful replication and activity throughout digestion. This study advances phage therapy research by demonstrating that a two-phage cocktail can selectively eliminate *E. cloacae* while preserving the overall microbiota structure and functionality. Our findings offer a new perspective on the potential of phages for targeted therapies, contributing to the development of personalized treatments for intestinal disorders associated with metabolic diseases. Future studies should explore the long-term effects of phage therapy on host health and its possible integration into therapeutic strategies for microbiota-associated disorders.

## 4. Materials and Methods

### 4.1. Study Design

An approach involving the use of a bacteriophage cocktail as a treatment was implemented. The study was structured around the evaluation of two groups. The first group was designated as the native gut microbiota of individuals with obesity treated with the bacteriophage cocktail, and the second group was the gut microbiota of individuals with obesity enriched with *E. cloacae* ATCC 13047. Each group was addressed using a set of three bioreactors. These bioreactors represented the ascending colon (AC), transverse colon (TC), and descending colon (DC), respectively. The experimentation focused on the interaction of the gut microbiota in each large intestine region under specific treatment conditions, evaluating microbiological, metabolic, and genomic aspects.

### 4.2. Adaptation of the Microbiota in the Automatic Robotic Intestinal System

This ex vivo model of the gastrointestinal tract consists of five bioreactors that sequentially simulate the stomach, small intestine, and the three regions of the large intestine (AC, TC, and DC). They act as model systems that allow the recreation and control of spatial, temporal, and environmental conditions encountered by microorganisms in the intestinal lumen. This respects relevant in vivo parameters such as gastrointestinal tract morphology, digestive and biliary juices composition, residence times, and pH profiles.

### 4.3. Fecal Inoculum

The fecal inoculum was generously provided by Dr. Marisela González Ávila of the ex vivo laboratory of the Department of Medical and Pharmaceutical Biotechnology at the Center for Research and Assistance in Technology and Design of the State of Jalisco. This fecal inoculum originated from fecal samples obtained on 11 April 2018 from 16 male and female volunteers, and then pooled. To preserve the integrity of the original microbiota, it was ensured that none of the participants had a history of antibiotic treatment during the 6 months before the study or at the time of sample collection. Participants were instructed to abstain from consuming fermented foods during the week prior to sample collection. Additionally, they had an average body mass index of 32.6 ± 2.9 kg/m^2^.

The fecal microbiota was inoculated into bioreactors representing the AC, TC, and DC. The pH was adjusted according to the corresponding region of the large intestine (AC: pH 5.5–6, TC: pH 6–6.5, DC: pH 6.5–7) using a NaOH 3 M or HCl 0.5 M buffer solution as needed. Additionally, they were maintained at a constant temperature of 37 °C with continuous agitation (120 rpm). These bioreactors were allowed to stabilize for a week, with 10 mL of a multivitamin solution added daily.

### 4.4. Digestion of the Characteristic Diet of Individuals with Obesity in Bioreactors

**Figure 5:**
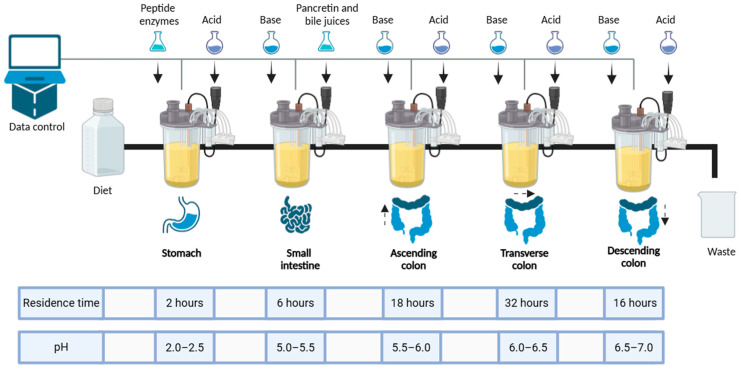
Schematic representation of the Automatic Robotic Intestinal System. Reactor 1: stomach; Reactor 2: small intestine; Reactors 3, 4, and 5 represent the ascending colon, transverse colon, and descending colon, respectively.

After completing the initial stabilization phase in the bioreactors using a multivitamin medium, the research progressed to the food digestion stage to achieve complete stabilization. The experimental design adopted was based on the methodological approach proposed by García-Gamboa et al. [21], specifically adapted to the needs and objectives of the present study (Figure 5).

The simulation began with the stomach phase, where 50 mL of a high calorie diet, composed of 2500 kcal with 50% carbohydrates, 20% proteins, and 30% lipids, was prepared. The pH was adjusted to a range of 2 to 2.5, and 0.125 g of pepsin was added to the system. This mixture was incubated at 37 °C with constant agitation at 120 rpm for two hours. The resulting digestion product (50 mL) was then transitioned to the small intestine simulation, where it was combined with a pancreatic enzyme solution containing 25,000 µg/L of lipase, 100,000 µg/L of amylase, and 60,000 µg/L of protease. The pH was adjusted from 5 to 5.5, and the mixture was maintained at 37 °C with constant agitation at 120 rpm for four hours. Subsequently, the digestion product (50 mL) was introduced into the bioreactor simulating the ascending colon, where the pH was adjusted to 5.5, and the system was incubated at 37 °C with agitation at 120 rpm for eighteen hours. The transverse colon simulation followed, during which 50 mL of the digestive product from the ascending colon was transferred to the transverse colon bioreactor. The pH was adjusted to 6.5, with conditions maintained at 37 °C and 120 rpm for thirty-two hours. Finally, the digestion mixture (50 mL) was introduced into the descending colon bioreactor. The necessary pH adjustments were made, and the agitation and temperature were sustained at 120 rpm and 37 °C for sixteen hours to complete the simulation process.

This digestive simulation process was cyclical, following a continuous scheme of three consecutive cycles. Each cycle had a total duration of 72 h. In the group with enriched gut microbiota from individuals with obesity, a prior enrichment was performed before the intervention with the bacteriophage cocktail in the AC, TC, and DC bioreactors through the inoculation of the *E. cloacae* ATCC 13047 strain. After a 72-h adaptation period, the bacteriophage cocktail treatment was implemented in both groups.

### 4.5. Characteristics of the Bacteriophage Cocktail and Its Inoculation

The bacteriophages that make up the cocktail under evaluation were isolated from wastewater in the state of Sinaloa, Mexico [49] with *E. cloacae* ATCC 13047 as the host bacteria. After inoculating the fecal microbiota into the bioreactors, 28 days were necessary for the microbiota to stabilize. Then, the first samples were collected at time 0, corresponding to the control period. Subsequently, the bacteriophage cocktail was inoculated in the AC for continuous digestion through the TC and DC. Thus, three interventions were completed with the bacteriophage cocktail in the three regions of the large intestine.

### 4.6. Bacteriophage and Bacteria Density in Bioreactor Samples

To determine the viral titer of the bacteriophage cocktail, a 2 mL sample was taken from each bioreactor. These samples were centrifuged at 10,000× *g* for 10 min at a temperature of 4 °C. After centrifugation, the supernatant was collected and placed in 1.5 mL tubes. The centrifugation process was repeated under the same conditions, and the supernatant was recovered using a 5 mL syringe. The supernatant was subjected to sequential filtration with pore-size membranes of 0.42 µm and 0.22 µm in 1.5 mL tubes to ensure adequate filtration. Decimal dilutions were prepared using a saline solution as the base. The spot test was then applied using the double-layer agar method described by Pallavali et al. [50]. To obtain the viral titer, the average number of lysis plaques generated was multiplied by the reciprocal of the dilution and the volume of inoculated bacteriophages. The experiment was performed in triplicate.

A 1 mL sample was taken from the bioreactors for counting enterobacteria, and decimal dilutions were prepared in peptone water. MacConkey agar medium was used to identify *Enterobacteriaceae*. They were inoculated with 10 µL of the dilution onto the plates. They were left to dry for at least 15 min at room temperature, followed by incubation for 24 h at 37 °C under aerobic conditions. The experiment was performed in triplicate. The results were analyzed using a repeated measures ANOVA with Bonferroni post hoc test in IBM SPSS Statistics 25 software.

### 4.7. Quantification of Short-Chain Fatty Acids

SCFAs were analyzed in bioreactor samples collected at the control and final time points following the inoculation of the phage cocktail. The samples were centrifuged at 13,500 rpm, filtered using syringe filters with a pore size of 0.45 µm, and transferred to HPLC vials for storage at 4 °C until analysis. Identification and quantification of SCFAs were performed using a WATERS HPLC system (Milford, MA, USA) [51], under the following conditions: an Aminex HPX-87H column (300 × 7.8 mm) (Bio-Rad, Hercules, CA, USA), 5 mM sulfuric acid as the mobile phase, an isocratic flow rate of 0.6 mL/min, a detector temperature of 55 °C, a run time of 30 min, a 1 mL aliquot size, and an injection volume of 10 µL. The detection wavelength was set at 210 nm to ensure precise identification and quantification.

The data were analyzed using IBM SPSS Statistics 25 software, applying the t-Student test for paired samples. Results are presented as the mean of three tests ± standard deviation, and statistical significance was set at *p* < 0.05.

### 4.8. DNA Extraction and 16S rRNA Metabarcoding Profile

The cetyltrimethylammonium bromide (CTAB) method with modifications was used for DNA extraction [52]. One millimeter of CTAB buffer was added to the sample tube, followed by vigorous vortexing. Subsequently, 700 µL was transferred to a new tube, and 700 µL of isoamyl chloroform (24:1) was added and vortexed, then allowed to rest for 5 min. The mixture was centrifuged at 12,300× *g* for 10 min. Two phases formed, the supernatant was taken and placed in a sterile tube, adding 500 µL of 2-propanol, and mixed by inversion, allowing it to rest for 2 min. After that time, it was centrifuged at 12,300× *g* for 5 min, and the supernatant was carefully decanted without losing the pellet. The pellet was washed by adding 500 µL of 70% ethanol, vortexed until the pellet separated, centrifuged at 12,300× *g* for 5 min, and the supernatant was carefully decanted without losing the pellet. The tube was left to dry at room temperature for 30 min and then in a thermoblock at 40 °C for 30 min. Finally, it was resuspended in nuclease-free sterile water. The concentration and purity of the DNA were determined using a NanoDrop 2000c spectrophotometer (Thermo Scientific, Wilmington, NC, USA). Additionally, DNA integrity was assessed by electrophoresis on a 1% agarose gel.

After DNA extraction, the variable V3 region of the 16S rRNA gene was amplified by PCR using a pair of primers, V3-338f and V3-533r [53]. The PCR products were sequenced in paired ends (300 cycles, 2 × 150 bp) on an Illumina Miniseq (San Diego, CA, USA).

### 4.9. Bioinformatic Analysis

Raw sequencing reads were preprocessed to ensure quality and accuracy. Sequencing adapters and low-quality sequences were removed, and paired-end reads were merged using fastp v0.23.4 [54]. Chimeric sequences were filtered from the assembled reads using vsearch v2.29.0 [55]. Taxonomic classification was performed using the Kraken2 v2.1.3 [56] against the SILVA132 database [57], while abundance estimations were refined using Bracken v2.9 [58]. To standardize data across samples, all datasets were rarefied to an equal number of sequences using vegan v2.6-8, and the rarefaction curves (Figure S1) were obtained in R v4.3.2 through RStudio v2024.09.0. The Kruskal–Wallis test was applied to identify significant differences in the relative abundance of taxonomic groups between treatments, and visualizations were created using the ggplot2 package in RStudio according to the metadata (Table S1). Alpha diversity was quantified using the Chao1 and Shannon indices through the vegan package, with statistical differences between intervention times evaluated via ANOVA followed by Tukey’s post hoc test. Beta diversity was assessed using non-metric multidimensional scaling (NMDS) based on Bray–Curtis dissimilarities, with microbial community composition differences statistically tested using PERMANOVA (permutational multivariate analysis of variance). Finally, to analyze the specific abundance of sequences belonging to *Enterobacter cloacae* in each sample, a BLAST v2.16.0 search was performed against the 16S rRNA gene of the *E. cloacae* ATCC 13047 strain using Geneious v2023.1 software. ANOVA followed by Tukey’s post hoc test was implemented in IBM SPSS Statistics 25 software to evaluate significant changes between intervention times in each colon region.

### 4.10. Statistical Analysis

Specific statistical tests and procedures were applied based on the data type and the corresponding methodology’s objectives. Details of the statistical methods, including significance thresholds, post hoc tests, and software used, are provided in the respective sections of the methodology. To account for multiple testing, the Benjamini–Hochberg method was applied across all analyses to control the false discovery rate.

## Figures and Tables

**Table 1:** Alpha diversity indexes of normalized intestinal microbiota of individuals with obesity before and after phage therapy intervention.

Colonic Regions	Index	Control	Mid Phage Therapy	Final Phage Therapy
Ascending	Chao1	23.67 ± 6.50 ^a^	27.33 ± 6.68 ^a^	18.33 ± 0.57 ^a^
	Shannon	1.67 ± 0.13 ^a^	1.87 ± 1.31 ^a^	1.57 ± 0.07 ^a^
Transverse	Chao1	24.50 ± 5.76 ^a^	32.41 ± 6.27 ^a^	23.67 ± 4.72 ^a^
	Shannon	1.59 ± 0.14 ^a^	1.72 ± 0.06 ^a^	1.66 ± 0.14 ^a^
Descending	Chao1	41.52 ± 15.51 ^a^	42.47 ± 4.10 ^a^	33.91 ± 3.39 ^a^
	Shannon	2.12 ± 0.13 ^a^	2.12 ± 0.07 ^a^	2.13 ± 0.06 ^a^

Values represent the mean of three tests ± standard deviation. Different letters indicate significant differences (*p* < 0.05) between intervention time points, as determined by ANOVA followed by Tukey’s post hoc test.

**Table 2:** Alpha diversity indexes of *Enterobacter*-enriched intestinal microbiota of individuals with obesity before and after phage therapy intervention.

Colonic Regions	Index	Control	Mid Phage Therapy	Final Phage Therapy
Ascending	Chao1	53.23 ± 5.06 ^a^	51.75 ± 13.66 ^a^	29.50 ± 5.89 ^b^
	Shannon	2.05± 0.07 ^ab^	2.35 ± 0.32 ^a^	1.60 ± 0.03 ^b^
Transverse	Chao1	44.97 ± 7.20 ^a^	43.29 ± 9.15 ^a^	34.08 ± 1.18 ^a^
	Shannon	1.62 ± 0.03 ^a^	1.60 ± 0.08 ^a^	1.82 ± 0.06 ^b^
Descending	Chao1	52.20 ± 8.25 ^a^	46.04 ± 4.82 ^a^	53.40 ± 5.7 ^a^
	Shannon	2.27 ± 0.01 ^a^	2.26 ± 0.05 ^a^	2.27 ± 0.02 ^a^

Values represent the mean of three tests ± standard deviation. Different letters indicate significant differences (*p* < 0.05) between intervention time points, as determined by ANOVA followed by Tukey’s post hoc test.

**Table 3:** *Enterobacter cloacae* 16S rRNA sequence count in enriched microbiota of individuals with obesity.

Intervention Time	Ascending Colon	Transverse Colon	Descending Colon
Control	7 ± 5.00 ^a^	79.67 ± 10.00 ^a^	18 ± 8.88 ^a^
Mid phage therapy	2.33 ± 1.63 ^ab^	28.67 ± 13.45 ^b^	12.17 ± 6.73 ^ab^
Final phage therapy	0.33 ± 0.57 ^b^	21.67 ± 7.63 ^b^	2.33 ± 1.52 ^b^

Values represent the mean of three tests ± standard deviation. Different letters indicate significant differences (*p* < 0.05) between the time of intervention in each colonic region, as determined by ANOVA followed by Tukey’s post hoc test.

## Data Availability

All relevant data are included in the article and its Supplementary Files. In addition, raw sequence data from this ex vivo assay were deposited in the NCBI Sequences Read Archive (SRA) under Bioproject accession number PRJNA1208567.

## Supplementary Materials

The following supporting information can be downloaded at: https://www.mdpi.com/article/10.3390/ijms26062805/s1.
