# Supplementary material for: A Two-Phage Cocktail Modulates Gut Microbiota Composition and Metabolic Profiles in an Ex Vivo Colon Model
Source: Int J Mol Sci. 2025 Mar 20;26(6):2805. doi: 10.3390/ijms26062805 (PMC11942677; doi:10.3390/ijms26062805)
Supplement: Supplementary file 1 [file ijms-26-02805-s001.zip › ijms-3529283-supplementary.pdf]

Supplementary material

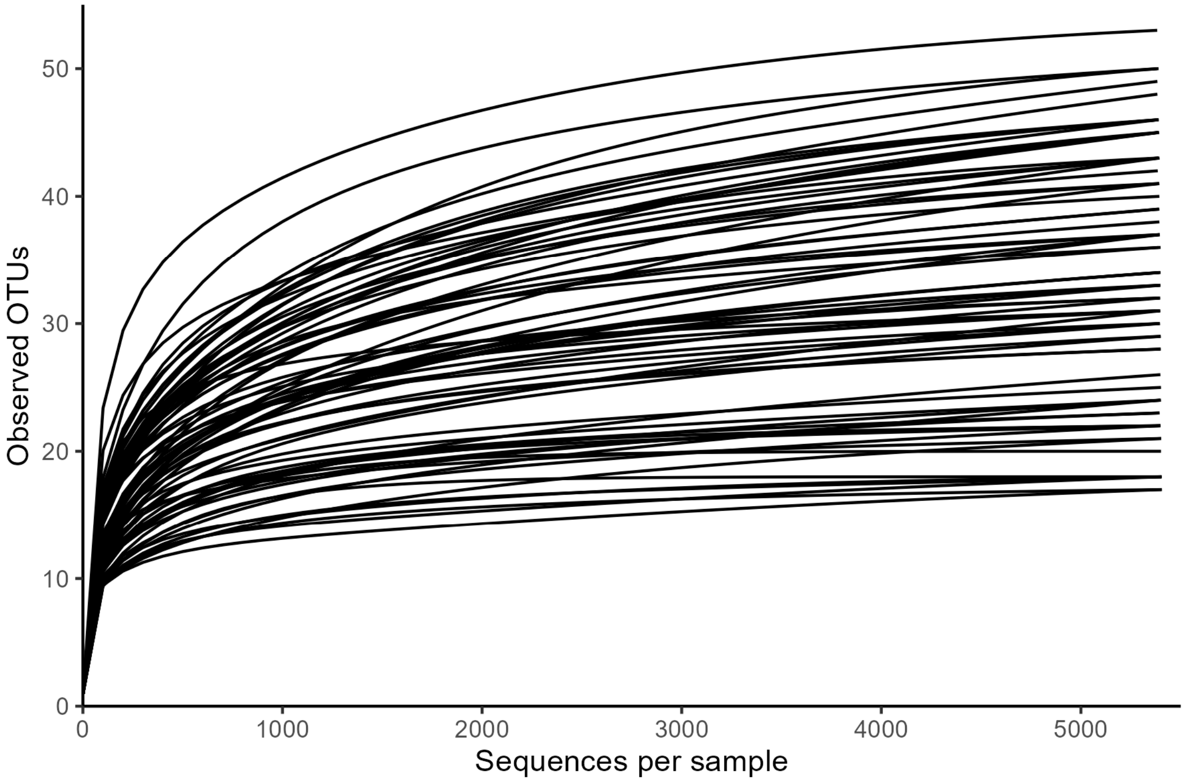

Figure S1. Rarefaction curves of all samples from ex vivo analyses.

Table S1. Metadata used for the metabarcoding profile analyses.

**Sample_ID**	**Type**	**Treatment**	**Colon**	**Time**
IA0_1	Non-enriched	Control	Ascendent	Time 0
IA0_2	Non-enriched	Control	Ascendent	Time 0
IA0_3	Non-enriched	Control	Ascendent	Time 0
IT0_1	Non-enriched	Control	Transverse	Time 0
IT0_2	Non-enriched	Control	Transverse	Time 0
IT0_3	Non-enriched	Control	Transverse	Time 0
ID0_1	Non-enriched	Control	Descendent	Time 0

ID0_2	Non-enriched	Control	Descendent	Time 0
ID0_3	Non-enriched	Control	Descendent	Time 0
IA1_1	Non-enriched	Mid phage therapy	Ascendent	Digestion 1
IA1_2	Non-enriched	Mid phage therapy	Ascendent	Digestion 1
IA1_3	Non-enriched	Mid phage therapy	Ascendent	Digestion 1
IT1_1	Non-enriched	Mid phage therapy	Transverse	Digestion 1
IT1_2	Non-enriched	Mid phage therapy	Transverse	Digestion 1
IT1_3	Non-enriched	Mid phage therapy	Transverse	Digestion 1
ID1_1	Non-enriched	Mid phage therapy	Descendent	Digestion 1
ID1_2	Non-enriched	Mid phage therapy	Descendent	Digestion 1
ID1_3	Non-enriched	Mid phage therapy	Descendent	Digestion 1
IA2_1	Non-enriched	Mid phage therapy	Ascendent	Digestion 2
IA2_2	Non-enriched	Mid phage therapy	Ascendent	Digestion 2
IA2_3	Non-enriched	Mid phage therapy	Ascendent	Digestion 2
IT2_1	Non-enriched	Mid phage therapy	Transverse	Digestion 2
IT2_2	Non-enriched	Mid phage therapy	Transverse	Digestion 2
IT2_3	Non-enriched	Mid phage therapy	Transverse	Digestion 2
ID2_1	Non-enriched	Mid phage therapy	Descendent	Digestion 2
ID2_2	Non-enriched	Mid phage therapy	Descendent	Digestion 2
ID2_3	Non-enriched	Mid phage therapy	Descendent	Digestion 2
IA3_1	Non-enriched	Final phage therapy	Ascendent	Digestion 3

IA3_2	Non-enriched	Final phage therapy	Ascendent	Digestion 3
IA3_3	Non-enriched	Final phage therapy	Ascendent	Digestion 3
IT3_1	Non-enriched	Final phage therapy	Transverse	Digestion 3
IT3_2	Non-enriched	Final phage therapy	Transverse	Digestion 3
IT3_3	Non-enriched	Final phage therapy	Transverse	Digestion 3
ID3_1	Non-enriched	Final phage therapy	Descendent	Digestion 3
ID3_2	Non-enriched	Final phage therapy	Descendent	Digestion 3
ID3_3	Non-enriched	Final phage therapy	Descendent	Digestion 3
EA0_1	Enriched	Control	Ascendent	Time 0
EA0_2	Enriched	Control	Ascendent	Time 0
EA0_3	Enriched	Control	Ascendent	Time 0
ET0_1	Enriched	Control	Transverse	Time 0
ET0_2	Enriched	Control	Transverse	Time 0
ET0_3	Enriched	Control	Transverse	Time 0
ED0_1	Enriched	Control	Descendent	Time 0
ED0_2	Enriched	Control	Descendent	Time 0
ED0_3	Enriched	Control	Descendent	Time 0
EA1_1	Enriched	Mid phage therapy	Ascendent	Digestion 1
EA1_2	Enriched	Mid phage therapy	Ascendent	Digestion 1
EA1_3	Enriched	Mid phage therapy	Ascendent	Digestion 1
ET1_1	Enriched	Mid phage therapy	Transverse	Digestion 1

ET1_2	Enriched	Mid phage therapy	Transverse	Digestion 1
ET1_3	Enriched	Mid phage therapy	Transverse	Digestion 1
ED1_1	Enriched	Mid phage therapy	Descendent	Digestion 1
ED1_2	Enriched	Mid phage therapy	Descendent	Digestion 1
ED1_3	Enriched	Mid phage therapy	Descendent	Digestion 1
EA2_1	Enriched	Mid phage therapy	Ascendent	Digestion 2
EA2_2	Enriched	Mid phage therapy	Ascendent	Digestion 2
EA2_3	Enriched	Mid phage therapy	Ascendent	Digestion 2
ET2_1	Enriched	Mid phage therapy	Transverse	Digestion 2
ET2_2	Enriched	Mid phage therapy	Transverse	Digestion 2
ET2_3	Enriched	Mid phage therapy	Transverse	Digestion 2
ED2_1	Enriched	Mid phage therapy	Descendent	Digestion 2
ED2_2	Enriched	Mid phage therapy	Descendent	Digestion 2
ED2_3	Enriched	Mid phage therapy	Descendent	Digestion 2
EA3_1	Enriched	Final phage therapy	Ascendent	Digestion 3
EA3_2	Enriched	Final phage therapy	Ascendent	Digestion 3
EA3_3	Enriched	Final phage therapy	Ascendent	Digestion 3
ET3_1	Enriched	Final phage therapy	Transverse	Digestion 3
ET3_2	Enriched	Final phage therapy	Transverse	Digestion 3
ET3_3	Enriched	Final phage therapy	Transverse	Digestion 3
ED3_1	Enriched	Final phage therapy	Descendent	Digestion 3

ED3_2	Enriched	Final phage therapy	Descendent	Digestion 3
ED3_3	Enriched	Final phage therapy	Descendent	Digestion 3
